# Inhibition of nitric oxide production in lipopolysaccharide-activated RAW 264.7 macrophages by Jeju plant extracts

**DOI:** 10.2478/v10102-009-0022-2

**Published:** 2009-12-28

**Authors:** Eun-Jin Yang, Eun-Young Yim, Gwanpil Song, Gi-Ok Kim, Chang-Gu Hyun

**Affiliations:** 1 Jeju Biodiversity Research Institute (JBRI), Seogwipo-si, Jeju 699-943, Korea; 2 Jeju Hi-Tech Industry Development Institute (HiDI), Ara-1-dong, Jeju 690-121, Korea

**Keywords:** cytotoxicity, inflammation, nitric oxide, plant extract

## Abstract

Nitric oxide (NO) produced in large amounts by inducible nitric oxide synthase (iNOS) is known to be responsible for the vasodilation and hypotension observed during septic shock and inflammation. Thus, inhibitors of iNOS may be useful candidates for the treatment of inflammatory diseases accompanied by the overproduction of NO. In this study, we prepared alcoholic extracts of Jeju plants and screened them for their inhibitory activity against NO production in lipopolysaccharide (LPS)-activated macrophages. Among the 260 kinds of plant extract tested, 122 extracts showed potent inhibitory activity towards NO production by more than 25% at a concentration of 100 µg/mL. Plants such as *Malus sieboldii*, *Vaccinium oldhamii*, *Corylus hallaisanensis*, *Carpinus laxiflora*, *Styrax obassia*, and *Securinega suffruticosa* showed the most potent inhibition (above 70%) at a concentration of 100 µg/mL. The cytotoxic effects of the plant extracts were determined by colorimetric MTT assays and most plant extracts exhibited only moderate cytotoxicity at 100 µg/mL. Therefore, these plants should be considered promising candidates for the further purification of bioactive compounds and would be useful for the treatment of inflammatory diseases accompanying overproduction of NO.

## Introduction

Nitric oxide (NO), which is synthesised by nitric oxide synthase (NOS) from L-arginine using NADPH and molecular oxygen, is a short-lived free radical and an intercellular messenger produced by a variety of mammalian cells, which include macrophages, neutrophils, platelets, fibroblasts, endothelium, neuronal, and smooth muscle cells. NO mediates a variety of biological actions ranging from vasodilatation, neurotransmission, inhibition of platelet adherence and aggregation, as well as the macrophage- and neutrophil-mediated killing of pathogens (Moncada *et al*., 1991, MacMicking *et al*., 1997; Oh *et al*., 2008). Chronic inflammation and infections lead to the up-regulation of a series of enzymes and signaling proteins in affected tissues and cells. The inducible forms of NOS are the most important pro-inflammatory enzymes responsible for increasing the levels of NO. Three isoforms of NOS have been identified and are classified into the following two major categories: constitutive and inducible. Expression of iNOS catalyses the formation of large amounts of NO, which plays a key role in the pathogenesis of a variety of inflammatory diseases. Therefore, the level of NO induced by iNOS may reflect the degree of inflammation and provides an indicator to assess inflammatory processes. Recently, several iNOS inhibitors have been reported as being isolated from plants such as 4-O-methylhonokiol (Oh *et al*., 2009), fraxinellone (Kim *et al*., 2009), 6-gingerol (Lee *et al*., 2009), tanshinone IIA (Fan *et al*., 2009), and arctigenin (Zhao *et al*., 2009). In addition, most of the inhibitory activity of these compounds towards NO production has been demonstrated to be through the inhibition of iNOS expression.

Plants have formed the basis of sophisticated traditional medicine systems that have been in existence for thousands of years across the globe. These plant based medicinal systems continue to play an essential role in health care today, and it has been estimated by the World Health Organization that approximately 80% of the world's inhabitants rely mainly on traditional medicines for primary health care (Cragg and Newman, 2008; Hsieh *et al*., 2008). Owing to its unique ecosystem, Jeju Island is famous for the richness and diversity of its flora with over 7800 species classified to date. Over the past few years, we have systematically evaluated and characterised selected plant species for their putative bioactivities or potential medicinal applications. In order to find new iNOS inhibitors from endemic Jeju plants, we have established a screen for the inhibitory activity towards NO production by measuring its production in LPS-stimulated RAW 264.7 cells.

## Materials and methods

### Plant materials and solvent extraction

The plants were collected from Jeju Island, Korea from 2006 to 2008. Voucher specimens were deposited at the herbarium of Jeju Biodiversity Research Institute. Verification of vouchers or living plants was performed by Dr. Gwanpil Song. Plant materials were air-dried, ground and extracted three times with 80% ethanol at room temperature. After the sample was filtered through two layers of cheesecloth, the filtered cakes were extracted and filtered three more times to increase the extraction yield. The filtrates were concentrated under reduced pressure, freeze-dried, and stored in a closed container until testing.

### Cell culture

Murine macrophage RAW 264.7 cells were purchased from the Korean Cell Line Bank (Seoul, Korea). They were cultured in Dulbecco's modified Eagle's medium (DMEM) containing 2 mM glutamine, 10 mM 4-[2-hydroxyethyl]-1-piperazineethanesulfonic acid (HEPES), penicillin (100 units/mL), streptomycin (100 µg/mL) and 10% foetal bovine serum. Cells were cultured at 37 °C in a humidified incubator with an atmosphere of 5% CO_2_.

### MTT assay for cell viability

3-(4,5-dimethylthiazol-2-yl)-2,5-diphenyltetrazolium bromide (MTT) is a pale yellow substrate that is reduced by living cells to yield a dark blue formazan product. This process requires active mitochondria, and only freshly dead cells do not reduce significant amounts of MTT. RAW 264.7 cells were cultured in 96-well plates for 18 hr, followed by treatment with LPS (1 µg/mL) in the presence of plant extracts at concentrations of 100 µg/mL. After a 24 hr incubation, MTT was added to the medium for 4 hr. Finally, the supernatant was removed and the formazan crystals were dissolved in dimethyl sulfoxide (DMSO). Absorbance was measured at 540 nm. The percentage of dead cells was determined relative to the control group.

### Nitric oxide assay

The nitric oxide assay was performed as described previously with slight modification (Yoon *et al*., 2009). After pre-incubation of RAW 264.7 cells (1.5 × 10^5^ cells/mL) with LPS (1 µg/mL) for 24 h, the quantity of nitrite in the culture medium was measured as an indicator of NO production. Amounts of nitrite, a stable metabolite of NO, were measured using Griess reagent (1% sulfanilamide and 0.1% naphthylethylenediamine dihydrochloride in 2.5% phosphoric acid). Briefly, 100 µL of cell culture medium was mixed with 100 µL of Griess reagent. Subsequently, the mixture was incubated at room temperature for 10 min and the absorbance at 540 nm was measured in a microplate reader. Fresh culture medium was used as a blank in every experiment. The quantity of nitrite was determined from a sodium nitrite standard curve.

## Results and discussion

In murine macrophage RAW 264.7 cells, LPS stimulation alone has been demonstrated to induce iNOS transcription and its protein synthesis, with a corresponding increase in NO production. Furthermore, LPS stimulation has also been shown to induce IκB proteolysis and NF-κB nuclear translocation (Xie *et al*., 1994; Henkel *et al*., 1993). Therefore, this cell system is an excellent model for drug screening and the subsequent evaluation of potential inhibitors against iNOS and NO production. In our search for natural products with anti-inflammatory activity, we prepared 80% ethanol crude extracts of 260 native plants from Jeju Island, Korea. All the plant samples were dissolved in 80% ethanol and diluted with sterile water to normalise the concentration of the test sample. The Griess reaction, a spectrophotometric determination for nitrite, was carried out to quantify the nitrite levels in the conditioned medium of RAW 264.7 cells treated with LPS. The final concentration of ethanol in the culture media was 0.1% and this concentration of ethanol did not show any effect on the assay systems. Table 1 shows the inhibitory activity by plant extracts towards NO production by LPS-activated macrophages. Of the 260 kinds of extracts, 122 extracts showed greater than 25% inhibition of NO production at the concentration of 100 µg/mL in the culture media. Among these 122 extracts, *Acer pictum*, *Viburnum dilatatum*, *Melia azedarach*, *Lonicera japonica*, *Osmunda japonica*, *Alnus firma*, *Lindera erythrocarpa*, *Platycarya strobilacea*, *Rhododendron werrichii*, *Weigela subsessilis* (, *Salix koreensis*, *Magnolia kobus*, *Corylus sieboldiana*, *Cornus walteri*, *Ulmus parvifolia*, *Morus bombycis*, *Aria alnifolia*, *Neoshirakia japonica*, *Actinodaphne lancifolia*, *Triadica sebifera*, *Elaeagnus umbellata*, *Oenothera glazioviana*, *Ficus erecta* var. *sieboldii*, *Rubus buergeri*, *Orixa japonica*, and *Cnidium japonicum* showed the most potent inhibition (greater than 70% inhibition) at the concentration of 100 µg/mL. The numbers of viable activated macrophages were not significantly altered by the plant extracts as determined by MTT assays, thereby indicating that the inhibition of NO synthesis by the plant extracts was not simply due to cytotoxic effects. Although some plant extracts such as *Idesia polycarpa*, *Artemisia scoparia*, and *Elsholtzia splendens* also exhibited potent inhibition (above 70%) towards NO synthesis at 100 µg/mL, there were some cytotoxic effects.

**Table 1:** Nitric oxide inhibition and cytotoxicity of Jeju plant extracts.

Scientific names	Parts	NO inhibition (%)	Cell viabilities (%)
*Carpinus laxiflora* (Siebold & Zucc.) Blume	L	80.42	108.36
*Elsholtzia splendens* Nakai ex F. Maek.	R	79.42	87.42
*Artemisia scoparia* Waldst. & Kit.	E	76.31	85.84
*Securinega suffruticosa* (Pall.) Rehder	L	74.38	100.77
*Styrax obassia* Siebold & Zucc.	L	73.50	110.63
*Malus sieboldii* (Regel) Rehder in Sarg*.*	L	72.08	104.36
*Idesia polycarpa* Maxim.	L	71.69	80.63
*Corylus hallaisanensis* Nakai	L	70.49	105.38
*Vaccinium oldhamii* Miq.	L	70.02	103.34
*Elaeagnus umbellata* Thunb. in Murray	L	69.69	99.02
*Carpinus turczaninowii* Hance	L	69.55	80.52
*Corylus sieboldiana* Blume	L	69.43	105.09
*Cudrania tricuspidata* (Carriere) Bureau ex Lavallee	L	69.08	79.81
*Triadica sebifera* (L.) Small	L	68.20	118.06
*Ternstroemia gymnanthera* (Wight & Arn.) Sprague	L	68.06	88.79
*Neoshirakia japonica* (Siebold & Zucc.) Esser	L	67.86	123.40
*Melia azedarach* L.	L	66.95	103.00
*Toxicodendron succedaneum* (L.) Kuntze	L	66.29	85.71
*Fraxinus mandshurica* Rupr.	L	66.09	84.82
*Euscaphis japonica* (Thunb.) Kanitz	E	66.03	81.50
*Sorbus commixta* Hedl.	L	64.85	89.00
*Lonicera japonica Thunb*. in Murray	E	64.39	91.72
*Malus sieboldii* (Regel) Rehder in Sarg.	L	64.11	82.17
*Ligustrum obtusifolium* Siebold & Zucc.	L	63.57	88.80
Osmunda japonica Thunb.	E	62.58	110.31
*Aria alnifolia* (Siebold & Zucc.) Decne.	L	62.30	91.69
*Orixa japonica* Thunb.	L	62.06	96.65
*Vaccinium bracteatum* Thunb. in Murray	ST	61.90	88.10
*Acer pictum* Thunb. in Murray var. *mono* (Maxim.) Maxim. ex Franch.	L	61.74	97.59
*Salix koreensis* Andersson in DC.	L	61.54	91.88
*Sorbus alnifolia* for. *hirtella* (Nakai) W. T. Lee	L	61.15	78.44
*Magnolia kobus* DC.	L	61.09	108.60
*Cornus walteri* Wangerin	L	60.08	137.70
*Oenothera glazioviana* Micheli in Mart.	L	56.61	110.47
*Actinodaphne lancifolia* (Siebold & Zucc.) Meisn. in DC.	L	56.03	96.52
*Lindera erythrocarpa* Makino	L	56.00	92.46
*Weigela subsessilis* (Nakai) L. H. Bailey	L	55.37	114.86
*Morus bombycis* Koidz.	L	54.93	93.72
*Ficus erecta* Thunb. var. *sieboldii* (Miq.) King	S	54.58	117.20
*Alnus firma* Siebold & Zucc.	L	54.31	102.81
*Rubus buergeri* Miq.	L	54.17	103.47
*Ulmus parvifolia* Jacq.	L	53.66	96.03
*Viburnum dilatatum* Thunb. in Murray	L	53.45	109.14
*Platycarya strobilacea* Siebold & Zucc.	L	51.98	113.98
*Cnidium japonicum* Miq.	E	50.55	95.48
*Rhododendron werrichii* Maxim.	L	50.09	109.39
Prunus serrulata var. quelpaertensis Uyeki	L	49.30	94.16
*Wisteria floribunda* (Willd.) DC.	L	49.11	94.32
*Broussonetia papyrifera* (L.) Lher. ex Vent.	L	48.93	115.46
*Viburnum furcatum* Blume ex Maxim.	L	48.73	92.97
*Sedum* bulbiferum Makino	E	48.70	100.85
*Eleutherococcus* gracilistylus (W. W. Sm.) S. Y. Hu	B	48.29	82.25
*Viburnum erosum* Thunb. in Murray	L	48.28	114.04
*Equisetum ramosissimum* Desf.	ST	48.13	125.83
*Weigela florida* (Bunge) A. DC. for. *subtricolor* Nakai	L	47.73	121.97
*Toxicodendron trichocarpum* (Miq.) Kuntze	L	47.69	111.54
*Ligustrum lucidum* W. T. Aiton	L	47.65	91.17
*Aster spathulifolius* Maxim.	E	47.58	101.82
*Cornus controversa* Hemsl.	L	46.82	104.47
*Kadsura japonica* (L.) Dunal	ST	46.27	89.71
*Euphorbia supina* Raf.	E	46.00	123.65
*Maackia fauriei* (H. Lev.) Takeda	L	45.82	91.89
*Zanthoxylum ailanthoides* Siebold & Zucc.	L	45.69	80.10
*Angelica dahurica* (Fisch. ex Hoffm.) Benth. & Hook. f. ex Franch. & Sav.	E	45.68	103.48
*Artemisia japonica* Thunb. in Murray	E	45.61	89.61
*Scutellaria strigillosa* Hemsl.	L	44.96	111.84
*Rhus javanica* L.	L	44.82	120.04
*Machilus japonica* Siebold & Zucc.	L	44.40	121.73
*Rhus javanica* L.	L	43.56	94.85
*Callicarpa japonica* Thunb. in Murray	L	42.60	110.03
*Abelia mosanensis* T. H. Chung	L	42.36	89.49
*Mallotus japonicus* (L. f.) Mull. Arg.	L	42.14	106.12
*Ginkgo biloba* L.	L	41.77	98.95
*Ficus oxyphylla* Miq. ex Zoll.	F	41.64	104.87
Staphylea bumalda DC.	L	41.39	91.47
*Styrax japonicus* Siebold & Zucc.	L	41.17	117.18
*Staphylea bumalda* DC.	L	40.29	115.05
*Akebia quinata* (Thunb.) Decne.	L	39.70	82.80
*Myrica rubra* Siebold & Zucc.	L	38.28	104.33
*Eucommia ulmoides* Oliv.	L	37.89	112.59
*Quercus dentata* Thunb. in Murray	L	37.44	95.14
*Euonymus* bungeana Maxim.	L	36.06	125.94
*Diospyros lotus* L.	L	35.76	104.78
*Limonium tetragonum* (Thunb.) Bullock	E	34.69	114.92
*Picrasma quassioides* (D. Don) Benn	L	34.48	104.93
*Tilia taquetii* C. K. Schneid.	L	33.94	116.22
*Prunus maxinowiczii* Rupr.	L	33.55	112.32
*Oenothera stricta* Ledeb.	E	33.54	112.38
*Trifolium pratense* L.		33.27	86.89
*Illicium anisatum* L.	L	33.17	109.30
*Sapindus mukorossi* Gaertn.	L	33.14	100.98
*Sophora japonica* L.	L	32.52	124.96
*Caesalpinia decapetala* (Roth) Alston	L	32.33	97.99
*Rubus parvifolius* L.	L	31.66	106.56
*Euonymus alatus* (Thunb.) Siebold	L	31.40	99.33
*Camellia japonica* L.	L	31.20	118.56
*Oenothera glazoviana* Micheli in Mart.	S	31.02	95.30
*Veratrum patulum* Loes.	E	30.88	109.28
*Taxillus yadoriki* (Siebold ex Maxim.) Danser	L	30.41	126.76
*Plantago asiatica* L.	E	30.35	117.34
*Callicarpa japonica* Thunb. in Murray	L	30.01	98.02
*Morus alba* L.	L	29.83	98.55
*Sambucus sieboldiana* (Miq.) Blume ex Graebn.	L	29.57	136.28
*Suaeda glauca* (Bunge) Bunge	E	28.73	106.04
*Xylosma congesta* (Lour.) Merr.	L	28.63	122.50
*Euonymus alatus* (Thunb.) Siebold	L	28.08	112.45
*Angelica dahurica* (Fisch. ex Hoffm.) Benth. & Hook. f. ex Franch. & Sav.	S	28.06	101.88
*Eleutherococcus gracilistylus* (W. W. Sm.) S. Y. Hu	L	27.80	90.93
*Celtis jessoensis* Koidz.	L	27.40	93.68
*Paederia scandens* (Lour.) Merr.	ST	27.17	108.00
*Euphorbia helioscopia* L.	E	27.13	91.69
*Clerodendrum trichotomum* Thunb.	L	26.93	123.13
*Asarum maculatum* Nakai	E	26.78	108.73
*Ampelopsis* brevipedunculata (Maxim.) Trautv.	E	26.67	104.98
*Lamium purpureum* L.	E	26.49	109.88
*Musa basjoo* Siebold & Zucc.	S	26.48	99.09
*Actinidia polygama* (Siebold & Zucc.) Maxim.	E	26.45	103.76
*Broussonetia papyrifera* (L.) Lher. ex Vent.	L	26.28	102.04
*Aesculus turbinata* Blume	L	26.20	117.06
*Angelica japonica* A. Gray	E	25.70	118.27
*Salsola komarocii* Iljin	E	25.64	99.39
*Cerastium glomeratum* Thuill.	E	25.03	105.92

Abbreviations: Entire plants (E), Roots (R), Stems and Twigs (ST), Leaves (L)

To conclude, these data suggest that extracts from the plant species examined in this study deserve further investigation in order to isolate the bioactive secondary metabolites with anti-inflammatory properties. Currently, experiments are in progress to analyse the active fractions from the extracts in order to determine the chemical structure of those compounds and to perform more extensive biological evaluations. Many compounds from medicinal plants have been demonstrated as inhibitors of the expression of iNOS in LPS-activated macrophages. Their structures can be categorised as sesquiterpene (Reddy *et al*., 2006; Choi *et al*., 2009), flavonoid (Chen *et al*., 2008; Paoletti *et al*., 2009), polyacetylenes (Kim *et al*., 2003), and lignans (Kim *et al*., 2008). Thus, plants demonstrating inhibitory activities against NO production will be promising candidates for the activity-guided isolation of active components exhibiting iNOS inhibitory activity, which may have therapeutic potential for the treatment of inflammation accompanying overproduction of NO. Further investigations are underway to characterise the active constituents present in these plant extracts.
